# The initiation of puberty in Atlantic salmon brings about large changes in testicular gene expression that are modulated by the energy status

**DOI:** 10.1186/s12864-019-5869-9

**Published:** 2019-06-11

**Authors:** Diego Crespo, Jan Bogerd, Elisabeth Sambroni, Florence LeGac, Eva Andersson, Rolf B. Edvardsen, Elisabeth Jönsson Bergman, Björn Thrandur Björnsson, Geir Lasse Taranger, Rüdiger W. Schulz

**Affiliations:** 1 Division Developmental Biology, Department Biology, Science Faculty, Reproductive Biology Group, Utrecht University, Kruyt Building, room O 806, Padualaan 8, 3584 CH Utrecht, The Netherlands; 2 INRA, UPR 1037 Laboratory of Fish Physiology and Genomics (LPGP), BIOSIT, OUEST-genopole, Bât. 16, Campus de Beaulieu, 35042 Rennes CEDEX, France; 3 Research Group Reproduction and Developmental Biology, Institute of Marine Research, Bergen, Norway; 4 Fish Endocrinology Laboratory, Department of Biological and Environmental Sciences, University of Gothenburg, S-40590 Gothenburg, Sweden

**Keywords:** Puberty, Androgens, Nutrition, Spermatogenesis, Testis, Transcriptomics

## Abstract

**Background:**

When puberty starts before males reach harvest size, animal welfare and sustainability issues occur in Atlantic salmon (*Salmo salar*) aquaculture. Hallmarks of male puberty are an increased proliferation activity in the testis and elevated androgen production. Examining transcriptional changes in salmon testis during the transition from immature to maturing testes may help understanding the regulation of puberty, potentially leading to procedures to modulate its start. Since differences in body weight influence, via unknown mechanisms, the chances for entering puberty, we used two feed rations to create body weight differences.

**Results:**

Maturing testes were characterized by an elevated proliferation activity of Sertoli cells and of single undifferentiated spermatogonia. Pituitary gene expression data suggest increased Gnrh receptor and gonadotropin gene expression, potentially responsible for the elevated circulating androgen levels in maturing fish. Transcriptional changes in maturing testes included a broad variety of signaling systems (e.g. Tgfβ, Wnt, insulin/Igf, nuclear receptors), but also, activation of metabolic pathways such as anaerobic metabolism and protection against ROS. Feed restriction lowered the incidence of puberty. In males maturing despite feed restriction, plasma androgen levels were higher than in maturing fish receiving the full ration. A group of 449 genes that were up-regulated in maturing fully fed fish, was up-regulated more prominently in testis from fish maturing under caloric restriction. Moreover, 421 genes were specifically up-regulated in testes from fish maturing under caloric restriction, including carbon metabolism genes, a pathway relevant for nucleotide biosynthesis and for placing epigenetic marks.

**Conclusions:**

Undifferentiated spermatogonia and Sertoli cell populations increased at the beginning of puberty, which was associated with the up-regulation of metabolic pathways (e.g. anaerobic and ROS pathways) known from other stem cell systems. The higher androgen levels in males maturing under caloric restriction may be responsible for the stronger up-regulation of a common set of (449) maturation-associated genes, and the specific up-regulation of another set of (421) genes. The latter opened regulatory and/or metabolic options for initiating puberty despite feed restriction. As a means to reduce the incidence of male puberty in salmon, however, caloric restriction seems unsuitable.

**Electronic supplementary material:**

The online version of this article (10.1186/s12864-019-5869-9) contains supplementary material, which is available to authorized users.

## Background

The brain-pituitary system of vertebrates has evolved as the main endocrine system regulating puberty and adult reproduction. Fish share the basic building blocks of the brain-pituitary-gonad axis with other vertebrates [1–3], but a number of genes critical for mammalian fertility are dispensable for piscine fertility. For example, the loss of neuropeptides like Kiss or Gnrh does not compromise fertility in fish, suggesting that systems regulating pituitary gonadotropin release operate in parallel and can compensate for the loss of single factors [4]. Also loss of Lh or its cognate receptor does not compromise pubertal development or spermatogenesis in zebrafish (*Danio rerio*) [5–7]. Surprisingly, androgens seem dispensable for spermatogenesis in fish as well. After losing the enzyme required for androgen production, secondary sex characteristics and reproductive behavior disappear but spermatogenesis continues normally in zebrafish or medaka (*Oryzias latipes*) [8, 9]. Nevertheless, loss of the androgen receptor does reduce testis weight in zebrafish, although some functional sperm are still produced [10, 11]. These observations differ from the situation in mice, where spermatogenesis fails in the absence of Lh or androgen signaling [12, 13], unless rescued by strong FSH receptor activation, which increases transcript levels of several genes also regulated by androgens [14]. While androgens potently stimulate spermatogenesis also in fish [10, 11, 15, 16], the relative androgen independence of piscine spermatogenesis may reflect the biological activities of Fsh-regulated growth factors such as Insl3 and Wnt5a produced by Leydig cells [17, 18], or Amh and Igf3 produced by Sertoli cells [19–21]. Clearly, the two gonadotropins target testicular signaling systems that regulate vertebrate spermatogenesis, but our knowledge on the composition and functioning of these testicular signaling systems is far from complete [22].

Hallmarks of puberty are the activation of spermatogenesis and an increase in androgen production [23]. Under the influence of reproductive hormones, spermatogonial stem cells (SSC) change their activity from infrequent self-renewal to more frequent self-renewal and to differentiating divisions that provide progenitor cells eventually differentiating into spermatozoa. The self-renewal and differentiation activity occurs in a strictly regulated and balanced manner to avoid SSC depletion on the one hand, and stem cell tumors on the other hand (see [24] for a comprehensive review on SSC biology). Since puberty in male salmon in aquaculture facilities can result in animal welfare and sustainability problems (see below), we are particularly interested in the period when puberty is initiated, i.e. the period when SSC activity changes and androgen production increases. To further our understanding of the regulatory processes promoting testis maturation, we wanted to examine the transcriptional changes accompanying the start of puberty. To this end, we used an experimental model that reflects the natural regulatory input. Therefore we compared testis tissue samples collected during the initial steps of spermatogenesis.

In Atlantic salmon, the initiation of male puberty is associated with a switch from allometric to super-allometric testis growth starting after the winter solstice. This is accompanied by elevated pituitary *fshb* transcript and plasma androgen levels, as well as an increase in single cell proliferation activity, involving both Sertoli cells and single type A spermatogonia, the earliest germ cell generation [25, 26]. Studies on different salmonid species have shown that a larger body weight and/or a faster growth rate increases the chance of entering puberty (chinook salmon [27]; Atlantic salmon [28, 29]). However, it is not known how body weight influences the entry into puberty. This is relevant since in salmon aquaculture, the endocrine changes associated with male puberty trigger osmoregulatory changes that compromise performance (e.g. seawater adaptability) as well as behavioral changes, eventually leading to animal welfare problems, reduced growth and the potential loss of fish [30]. Therefore, we exposed fish to two feed rations that generated body weight differences. Our aim was to study transcriptional changes accompanying the initial steps of pubertal testis maturation, and to examine how the nutritional status affects these changes. Great care was taken to select testis tissue samples for microarray analysis. The selection was primarily based on analyzing testicular proliferation activity, but also included the quantification of circulating androgens and of selected pituitary and testis gene transcripts.

## Results

### Selection of samples for transcriptomic analyses

Testis samples containing groups of synchronously proliferating germ cells, next to single proliferating cells, or males with testes containing type B spermatogonia, were excluded from the microarray analysis. These individuals were considered as too far advanced in spermatogenesis (Additional file 1: Figure S1; progressed). All remaining males showed type A spermatogonia as the furthest developed germ cell type but differed as regards the single cell proliferation activity of spermatogonia and Sertoli cells (Additional file 1: Figure S1) and as regards the feed ration the fish received, establishing the groups NR_imm_ (normal ration immature) and RR_imm_ (restricted ration immature), showing low, and NR_mat_ (normal ration maturing) and RR_mat_ (restricted ration maturing), showing elevated single cell proliferation activity. Moreover, different from immature fish, maturing males showed spermatogenic tubuli containing Sertoli cells that were not in contact with germ cells (“free” Sertoli cells), resulting in an elevated number of Sertoli cells.

**Fig. 1:**
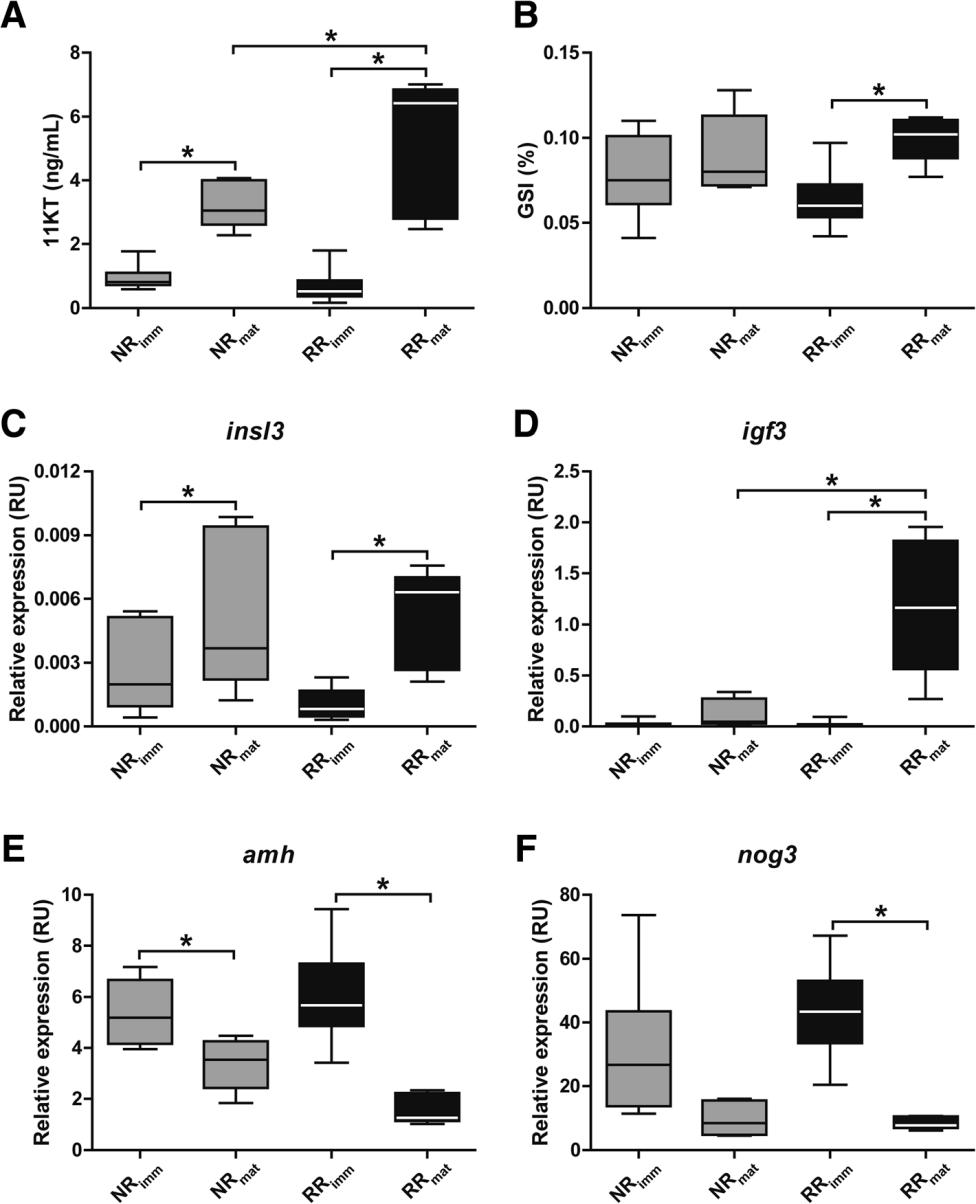
Characterization of the experimental groups. Androgen plasma levels (11KT, ng/mL) (**a**), gonadosomatic indices (**b**) and testicular levels of selected transcripts (**c-f**) analyzed in immature (imm) and maturing (mat) salmon receiving a normal feed ration (NR) or a restricted feed ration (RR). Data are expressed as mean ± SEM (*N* = 5–16; two-way ANOVA followed by Bonferroni’s test, * *P* < 0.05 at the α level of 0.05). RU, relative units

Quantification of plasma 11-ketotestosterone (11KT) levels, the main androgen in fish, confirmed the morphological analysis and showed that 11KT plasma levels were ~ 5-fold higher in maturing fish (Additional file 2: Table S1). Interestingly, RR_mat_ males showed significantly higher 11KT levels than NR_mat_ males (Fig. 1a). Also, the gonadosomatic index (GSI) was higher in maturing than immature males, but was only statistically significant in the RR_mat_ group (Fig. 1b). Similarly, quantifying selected pituitary gene transcripts showed that *fshb*, *lhb*, and *gnrhr4* mRNA levels were all significantly higher in RR_mat_ than in RR_imm_ males (Table 1), while these differences were not significant between NR_mat_ and NR_imm_ fish (Table 1).

**Table 1:** Influence of the ration regime, or the maturational status, on morphometric parameters and pituitary gene expression

	NR_imm_	NR_mat_	RR_imm_	RR_mat_
Weight (Kg)	3.84 ± 0.32^a^	5.42 ± 0.50^b^	2.28 ± 0.10^c^	2.99 ± 0.36^ac^
Length (cm)	65.00 ± 1.31^a^	70.60 ± 2.32^a^	58.38 ± 1.21^b^	63.40 ± 2.16^ab^
Visceral fat (%)	36.27 ± 1.13	38.10 ± 1.79	24.74 ± 1.54	nd
Muscle fat (%)	16.85 ± 0.57	19.68 ± 0.61	14.18 ± 0.38	nd
K	1.37 ± 0.05^a^	1.52 ± 0.03^b^	1.15 ± 0.04^bc^	1.11 ± 0.02^cd^
*fshb* (RU)	11.35 ± 2.01^a^	20.89 ± 5.31^ab^	4.10 ± 0.61^a^	31.06 ± 6.48^b^
*lhb* (RU)	22.38 ± 8.74^a^	39.97 ± 16.68^ab^	11.42 ± 4.78^a^	88.71 ± 34.28^b^
*gnrhr4* (RU)	2.35 ± 0.17^a^	3.39 ± 0.67^ab^	1.69 ± 0.12^a^	3.29 ± 0.38^b^

Statistical significance (*N* = 6–15; two-way ANOVA followed by Bonferroni’s test, * *P* < 0.05 at the α level of 0.05) is indicated by different letters. *RR* Restricted ration, *NR* Normal ration, *imm* immature, *mat* maturing, *K* condition factor, *RU* Relative units, *nd* not determined

The two feed rations resulted in differences as regards body weight (BW) and length (BL), visceral and muscle fat content, as well as the condition factor (Additional file 2: Table S1). Within the NR group, but not the RR group, maturing males were characterized by larger body weight and muscular fat content (Table 1). Unfortunately, the muscular and visceral fat samples from the 5 maturing males of the RR group were lost, so that a direct comparison to immature RR males is not possible. However, maybe not surprisingly, the levels in immature RR males were clearly lower than found in (immature and maturing) NR males.

In addition to morphometric parameters, plasma androgen and selected pituitary transcript levels that were used to corroborate the individual reproductive status, we also examined the testicular transcript levels of selected candidate genes before applying the full-scale transcriptome analysis. Growth factors known to stimulate spermatogonial proliferation in zebrafish, such as *insl3* and *igf3*, were higher in maturing than in immature males in both ration groups (Fig. 1c-d). The transcript levels of two genes functioning in the Tgfβ signaling system, on the other hand, *amh* (inhibiting spermatogenesis in eel and zebrafish) and *nog3* (a Bmp−/Activin-binding protein) followed the opposite expression pattern, being lower in maturing testes (Fig. 1e-f).

### Gene expression profiling in testis of pubertal salmon with different nutritional status

**Fig. 2:**
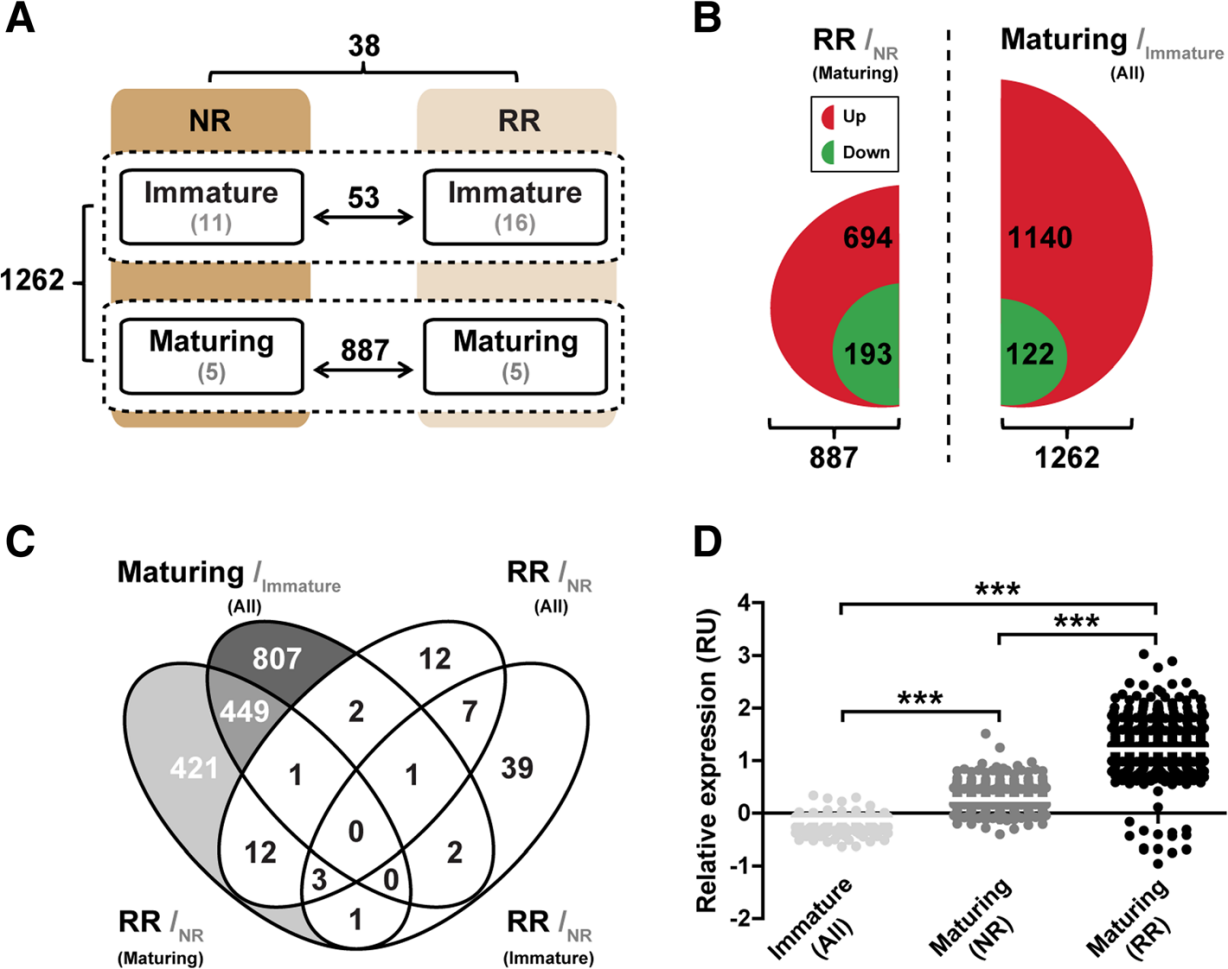
Transcriptomic response at puberty of Atlantic salmon testis tissue sampled from males fed a normal (NR) or restricted ration (RR; 43% of NR). **a** Total numbers of DEGs (*N* = 5–16, indicated by grey numbers; Student’s t-test, *P* < 0.01 at the α level of 0.05, FDR < 0.05; fold change > │1.5│) in each pairwise comparison. Numbers next to the brackets give the number of total DEGs. **b** Up- and down-regulated genes identified during male salmon maturation independently of the food regime (right panel), or exclusively in males exposed to the restricted ration (left panel). For each comparison, black and grey letters represent the test condition and its corresponding control, respectively. **c** Venn diagram representation of transcripts differentially expressed in all pairwise comparisons performed. For each comparison, black and grey letters represent the test condition and its corresponding control, respectively. **d** Stimulatory effect on testicular gene expression during puberty for a set of 449 shared genes (one-way ANOVA followed by Tukey’s test, *** *P* < 0.001 at the α level of 0.05), and modulation of the magnitude of this effect by the feed ration

The results show that assigning males to one of the four groups NR_imm_, NR_mat_, RR_imm_ or RR_mat_ is supported by selected markers for the morphological and physiological state of the tissue. To investigate energy status-related changes in testicular gene expression during the initiation of pubertal testis maturation, microarray analyses were performed using testis tissue collected from these four groups. Comparing immature males from the two ration groups revealed only a limited number of differentially expressed genes (DEGs, 53; Fig. 2a and Additional file 3: Table S2). However, a very high number of DEGs (887; Fig. 2a) were found when comparing maturing males from the two ration groups, ~ 78% of these transcripts being up-regulated (Fig. 2b and Additional file 3: Table S2). The most prominent difference in gene expression (1262 DEGs; Fig. 2a) was seen when comparing all maturing to all immature fish. Also in this data set a very high proportion of transcripts was up-regulated (~ 90%; Fig. 2b and Additional file 3: Table S2). On the contrary, only 38 DEGs were identified when comparing the two ration groups (Fig. 2a and Additional file 3: Table S2). Sets of modulated genes for the four comparisons described above were then compared using Venn diagrams. This approach showed that most of the shared DEGs between comparisons were found in testis samples from all maturing males, and RR_mat_ males (i.e. 449 DEGs; Fig. 2c). Most common genes followed the same pattern in the two data sets (up-regulated expression in all maturing and the RR_mat_ males; Additional file 4: Table S3). Thus, for this group of genes, the expression levels were low in immature fish, intermediate in NR_mat_ fish, and high in RR_mat_ fish; a statement applying to ~ 93% of the 449 DEGs (Additional file 4: Table S3). Hence, most DEGs were up-regulated in testis tissue during the initiation of puberty, and for a set of 449 genes, the magnitude of the change was significantly higher in the males maturing under caloric restriction.

**Fig. 3:**
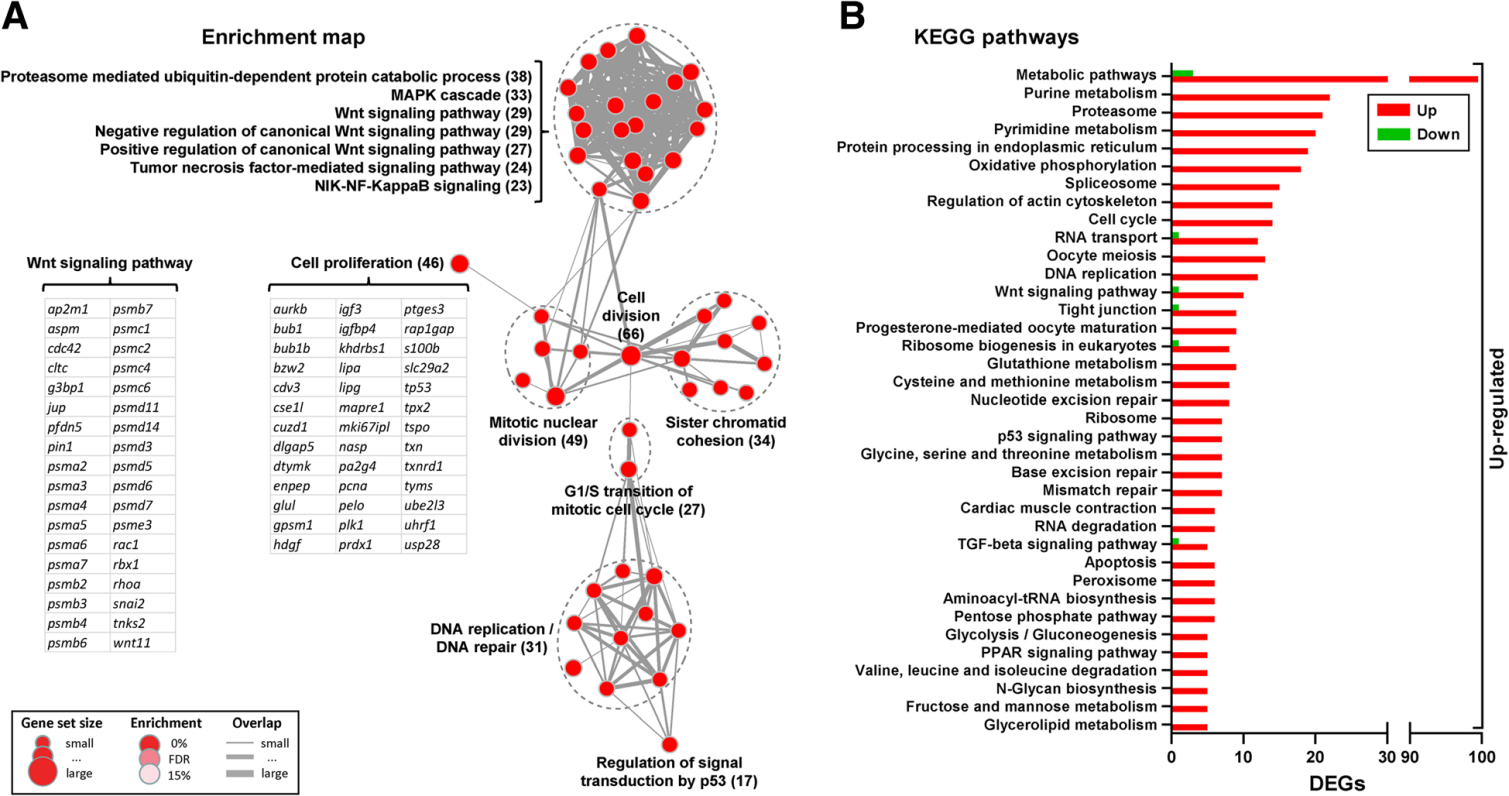
Functional enrichment of maturation-induced gene expression in the salmon testis. **a** The data set containing modulated genes at puberty (i.e. 1262) was mapped (after GO enrichment analysis) resulting in a network of functionally related gene sets (red nodes) that form enrichment groups. Nodes represent statistically significant GO terms (*P* < 0.005 at the α level of 0.05, FDR < 0.01, overlap < 0.5) and links (grey edges) indicate the number of overlapping genes (indicated by their thickness) between connected sets. Groups of closely related GO terms are encircled with dashed lines and labeled. Number of identified genes is shown in brackets. **b** KEGG pathways identified in the maturing salmon testis. Each pathway shown is represented by at least 5 DEGs (*P* < 0.01 at the α level of 0.05, FDR < 0.05) and has a ratio of regulated genes (up−/down-, or vice versa) higher than 5. DEGs are highlighted with red (up-) or green (down-regulated) background

To gain more insight into the biological significance of the transcriptomic changes in maturing males, the 1262 DEGs were tested and mapped for functional enrichment. Enrichment analysis showed numerous overlapping gene sets (genes belonging to Gene Ontology [GO] terms closely related) involved in the regulation of the Wnt signaling pathway (Fig. 3a) including genes important for vertebrate spermatogenesis, such as *aspm*, *pfdn5*, *pin1* and *snai2*. Activation of a number of genes related to cell proliferation was found (Fig. 3a), which fits the observation of an elevated frequency of pH 3-positive cell nuclei (Additional file 1: Figure S1; maturing). This set included transcripts expressed in the mammalian testis (*nasp*, *tp53*, *bub1*, *plk1*), as well as proliferation markers (*mki67ipl*, *pcna*) and growth factors (*igf3*, *hdgf*). We also identified a broad diversity of cell division-related genes (grouped as *Cell division*, *Mitotic nuclear division*, *G1/S transition of mitotic cell cycle*, *Sister chromatid cohesion*; Fig. 3a), including several cyclins (*ccna2*, *ccnb1*, *ccnb2*, *ccnb3*, *ccne1*), cyclin-dependent kinases (*cdkn2d*, *cdkn3*, *cks1b*) and cell division proteins (*cdc6*, *cdca7*, *cdk2*, *cdk10*) (Additional file 3: Table S2). In addition, we found a variety of different protein complexes important for chromosome structure/organization during cell division, such as condensins (*ncapd2*, *ncapd3*, *ncapg*), chromosome-associated proteins (*smc2*, *smc4*) and kinesins (*kif2c*, *kif15*) (Additional file 3: Table S2). In accordance with the stimulated expression of cell division- and proliferation-related genes, this functional analysis also identified enrichments in gene sets involved in DNA metabolism (Fig. 3a), mainly DNA replication (*mcm5*, *mcm7*, *rfc2*, *rfc4*) and repair (*rad51a*, *rad54b*, *fen1*, *top2a*) (Additional file 3: Table S2). Overall, the morphometric and proliferation data are well in line with the transcriptomic data as regards processes related to cell proliferation.

KEGG pathway analysis confirmed enrichments in similar gene sets as described above, but also provided additional information. KEGG analysis retrieved a variety of significantly enriched metabolic processes (Fig. 3b). The most highly represented KEGG term, labeled as *Metabolic pathways* (Fig. 3b), representing a total of 101 DEGs, included fatty acid- (*acsbg2*, *echs1*, *gpam*, *mecr*), lipid- (*agpat*, *lipg*, *cbsb*) and oxidative stress-related transcripts (*idh1*, *idh3a*, *aldoaa*) (Additional file 5: Table S4). Furthermore, increased expression of genes associated with *Purine* and *Pyrimidine metabolism*, as well as *Glycerolipid* and amino acid (*Glutathione*, *Cysteine* and *methionine*) *metabolism* was also observed in maturing males (Fig. 3b and Additional file 5: Table S4). Signaling pathways, such as Tgfβ (*bmp6*, *bmp7b*, *ndr1*) and Ppar (*fads2*, *fabp2*, *fabp3*, *lpl*), were also activated in the salmon testis.

**Fig. 4:**
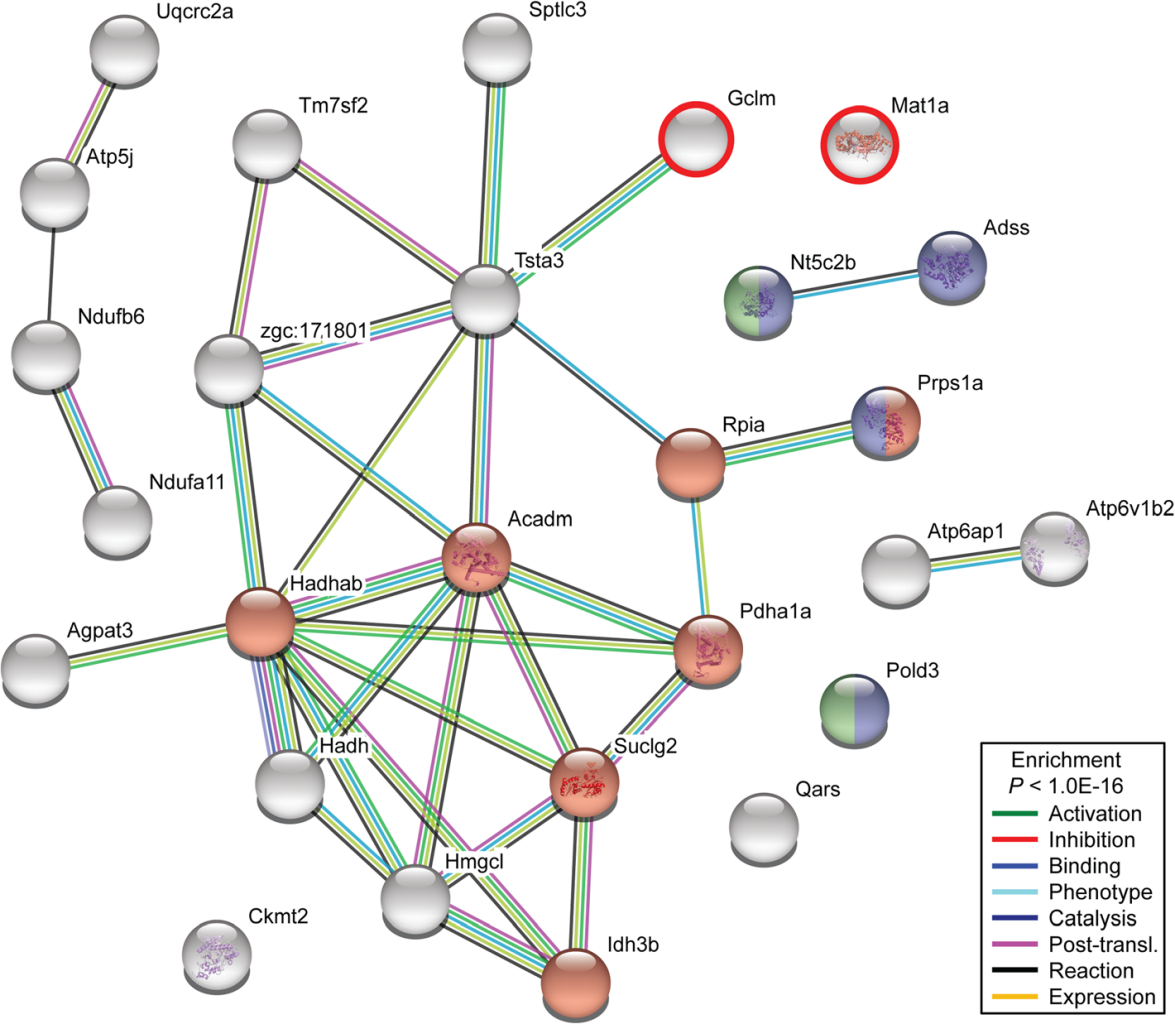
Protein-protein interaction map for candidates found in pubertal males exposed to the restricted feed ration. The interaction network was generated using STRING database v10.5 (default settings; medium confidence of 0.4). A total number of 27 candidate genes was considered for the analysis. Proteins are represented as nodes, and lines indicate associations based on known functional interactions in zebrafish (see right bottom corner). The network is significantly enriched in interactions (*P* < 1.0E-16 at the α level of 0.05, FDR < 0.05). Red, purple and green nodes indicate proteins involved in *Carbon*, *Purine* and *Pyrimidine metabolism*, respectively. Nodes encircled in red color represent additional *Carbon metabolism*-associated proteins known in mammals (but not yet in zebrafish)

Interestingly, half of the KEGG terms enriched in all maturing males were also retrieved using as input only the genes in common with the RR_mat_ males (i.e. 449 DEGs; Fig. 2b; Additional file 6: Figure S2B). However, only 3 KEGG pathways were found enriched in the data set containing the specific transcripts modulated exclusively in RR_mat_ males (i.e. 421 DEGs; Fig. 2b), and among those the KEGG term *Metabolic pathways* contained the majority of the genes identified (i.e. 27; Additional file 6: Figure S2C). Next, we investigated potential protein-protein interactions for these 27 candidates showing that 23 proteins encoded by their corresponding genes were functionally associated (Fig. 4). This analysis found interacting proteins in particular groups (Fig. 4; Additional file 6: Figure S2D), showing several proteins involved in *Carbon metabolism* (Prps1a, Rpia, Pdha1a, Suclg2, Idh3b, Acadm, Hadhab; red nodes in Fig. 4). Additional proteins also retrieved here, such as Gclm and Mat1a (encircled in red in Fig. 4), also participate in carbon metabolism in mammals but respective information is not available in fish yet. Finally, *Purine* and *Pyrimidine metabolism*-related proteins (purple and green nodes, respectively) were also identified (Fig. 4; Additional file 6: Figure S2D).

**Fig. 5:**
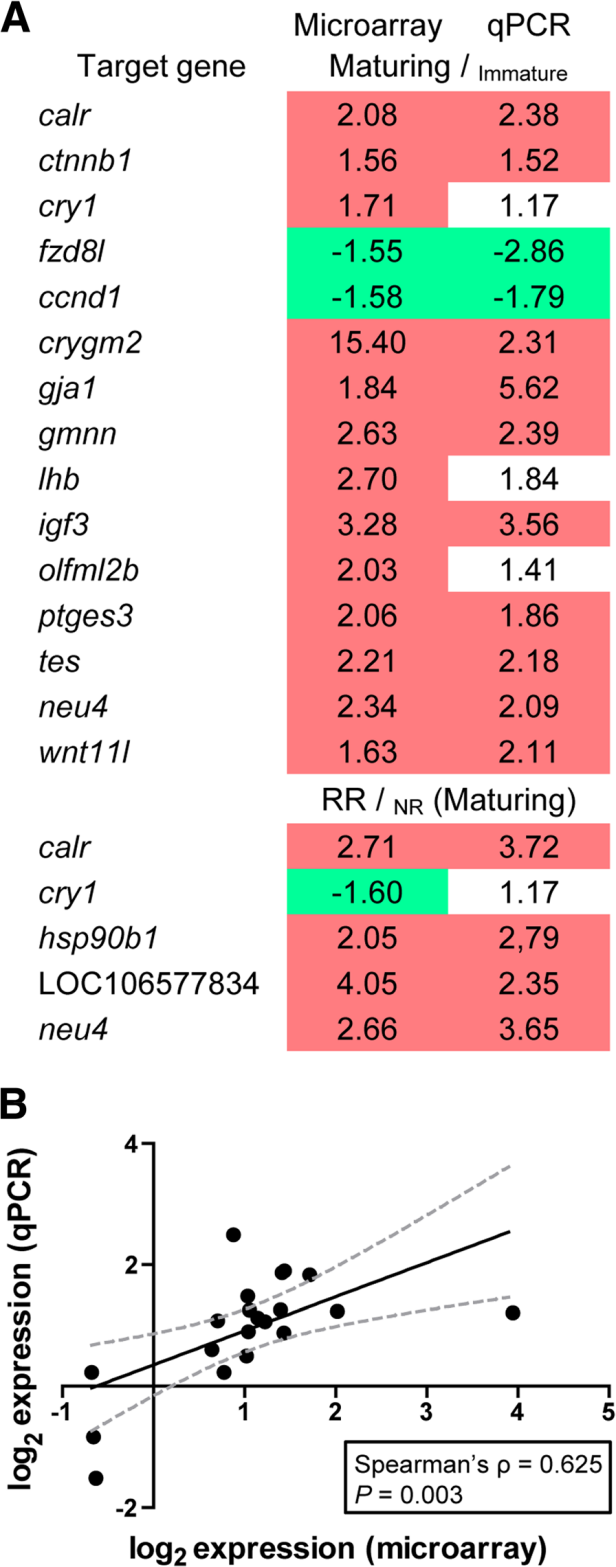
Validation of microarray results by qPCR analysis. **a** Data are expressed as fold change with respect to the control group (experimental condition appearing as subscripts). Statistical significance (*N* = 6–15; Student’s t-test, *P* < 0.05 at the α level of 0.05) is highlighted with red (up-) or green (down-regulated) background. RR, restricted ration; NR, normal ration. **b** Correlation of microarray and qPCR. Comparison of changes in gene expression (represented as log2) derived from qPCR and microarray hybridizations revealed a significant correlation (Spearman’s rank test, *P* = 0.003 at the α level of 0.05)

In order to confirm the microarray results, expression patterns of 17 selected genes were analyzed by qPCR using the same sample set employed for microarray hybridization. Except for *cry1* (and only when comparing ration effects in maturing males; i.e. RR/_NR_), results on transcript level changes obtained by both techniques were similar as regards the direction and magnitude (Fig. 5a). Comparison of changes in gene expression (represented as log2) showed a significant correlation (*P* = 0.003; Fig. 5b) among the data derived from qPCR and microarray hybridization.

## Discussion

The initiation of pubertal testis maturation was accompanied by an increased level of single cell (type A spermatogonia and Sertoli cells) proliferation and androgen production. Transcriptional changes accompanying these events included several signaling systems (e.g. Tgfβ, Wnt, insulin/Igf, nuclear receptors), but also the activation of metabolic pathways (e.g. anaerobic metabolism, protection against ROS). Pituitary gene expression data suggest that an increased gonadotropic stimulation is responsible for the elevated circulating androgen levels in maturing fish. While feed restriction lowered the incidence of puberty, those males maturing despite feed restriction showed higher plasma androgen levels compared to males maturing on normal ration. Likewise, a group of 449 genes was up-regulated more prominently in testes from fish maturing under caloric restriction than in testes of maturing fully-fed fish. However, in the maturing fully-fed fish, this group of 449 DEGs was still significantly up-regulated in comparison to immature males. Finally, 421 genes were specifically up-regulated in testes from fish maturing under caloric restriction. Here, enriched pathways retrieved included Carbon metabolism genes, a pathway relevant for nucleotide biosynthesis and for placing epigenetic marks.

Males with an increased germ and Sertoli cell proliferation activity also displayed elevated pituitary *fshb* transcript and plasma androgen levels. We did not have the possibility to quantify Fsh protein levels, but previous work in other salmonid species showed that plasma Fsh levels were elevated at the beginning of testis maturation [31, 32]. Since Fsh is a potent steroidogenic hormone in fish, based on Fsh receptor expression by Leydig cells [33–35], it was assumed that elevated plasma androgen levels closely reflected Fsh bioactivity, also bearing in mind that plasma Lh levels do not increase in salmonids until approaching the spawning season [31, 32]. Both Fsh [20, 36] and androgens [16, 37] trigger proliferation and changes in testicular gene expression [17, 38] in different fish species. We therefore relate the transition to increased single cell proliferation to Fsh- and/or androgen-mediated changes in testicular transcription, since Fsh and androgens both are major stimulators of spermatogenesis [15–17, 39, 40] and the onset of puberty [26, 41] in fish. This maturation-associated transcriptional network (1262 DEGs; see Fig. 3) included Wnt and Tgfβ signaling pathways as well as a broad diversity of genes related to cell division and metabolism. Previous studies on transcriptomic changes during fish spermatogenesis showed functional enrichments similar to those reported here [38, 42–45]. The regulation of spermatogenesis involves a similar set of signaling pathways as in mice [46, 47], suggesting that the global composition of this network is a conserved feature of vertebrate spermatogenesis regulation. It is interesting to note, however, that previous transcriptomic studies in fish, as well as in mice, covered a broader range of germ cell stages, often [38, 42, 43, 45–47] even the complete spermatogenic process. Our study revealed a similarly broad array of pathway activation, although we investigated only the initial step of pubertal spermatogenesis, the mitotic expansion of Sertoli cells and of undifferentiated type A spermatogonia and the associated increase in androgen production.

### Androgens

The transcriptional changes in the pubertal salmon testis are associated with elevated 11KT plasma levels, and the majority (90%) of the DEGs were up-regulated. This is similar to what has been observed in immature male rainbow trout (*Oncorhynchus mykiss*) following androgen treatments [48], while the androgen receptor antagonist flutamide increased the proportion of down-regulated genes in sexually mature zebrafish [49]. It seems that androgens predominantly activate gene expression in the fish testis. Androgen signaling is very important for spermatogenesis in mammals [50]. Similarly, androgens strongly stimulate spermatogenesis in fish [16, 40], which is supported by the observation that testis weight decreased ~ 5-fold after loss of the androgen receptor gene in zebrafish, although some sperm was still produced [10, 11]. However, loss of an enzyme required for androgen synthesis resulted in the loss of secondary sexual characters or reproductive behavior, but did not disrupt spermatogenesis in medaka or zebrafish [8, 9], suggesting that androgen-independent mechanisms are also involved in the regulation of spermatogenesis in fish. These mechanisms are probably related to Fsh-regulated growth factors such as Insl3 [51], Igf3 [20] and others [17, 38]. The set of 1262 DEGs (Fig. 3) retrieved from all maturing males represent genes responding to Fsh or androgens. Looking for androgen-specific genes, we searched a data set on androgen-induced changes in rainbow trout testicular gene expression in vivo [48]. Among the retrieved genes (see Additional file 7: Table S5), two candidates attracted our interest and are discussed further below, namely follistatin-like 1, that suggests a role for other Tgfβ family members, and a zinc transporter that suggests metabolic processes related to oxidative stress.

### Tgfβ signaling

Follistatin-like 1 (*fstl1*) is a gene similar to the Tgfβ binding protein follistatin, which restricts the biological activity of Tgfβ growth factors, such as activin. In the Japanese eel (*Anguilla japonica*), activin is up-regulated during hCG-induced spermatogenesis and recombinant activin stimulated the differentiating proliferation of spermatogonia [52, 53]. It is therefore possible that at the very initial stage of testis maturation, when self-renewal proliferation of type A spermatogonia increases the population of undifferentiated type A spermatogonia (see Additional file 1: Figure S1), the pro-differentiation activity of activin is modulated by the increased availability of follistatin-like 1. Interestingly, in immature rainbow trout, transcript levels of the related *fstl3* were up-regulated by Fsh, and those of the beta A subunit of activin (*inhba*) were down-regulated by androgens [38]; in both immature rainbow trout and adult zebrafish, *inhbab* transcript levels were up-regulated in response to Fsh when androgen production was blocked [17]. Future work should test the hypothesis that up-regulation of activin activity at the onset of testis maturation is initially tempered by the increased availability of binding proteins until the population of undifferentiated spermatogonia expanded sufficiently.

Another Tgfβ family member of interest was *bmp6*, showing increased transcript levels. In mice, BMP6 stimulated Sertoli cell proliferation [54], and Sertoli cells also proliferate in the maturing salmon testis (Additional file 1: Figure S1). Bmp signaling is also relevant for germ cells, supported by the observation that the loss of the Bmp receptor Alk6b in zebrafish impaired spermatogonial differentiation, leading to an accumulation of type undifferentiated type A spermatogonia [55]. Also *nog3* transcript levels decreased in maturing testicular tissue (Fig. 1f). Noggin is a TGFβ binding protein that in mammals functions as a BMP binding protein [56] and appears to have similar functions in zebrafish [57]. Finally, we found that anti-Mullerian hormone (*amh*) transcript levels decreased in maturing testicular tissue (Fig. 1e). This observation has been made in different species, and functional studies in the eel [19], medaka [58] and zebrafish [59] demonstrated different aspects of the inhibitory effects of Amh. In zebrafish, this included inhibiting the proliferation of undifferentiated type A spermatogonia, the differentiation of type A spermatogonia, androgen production, or *igf3* gene expression as well as Igf3 bioactivity [21, 60]. Depending on the species, *amh* transcript down-regulation by androgens [19] or by Fsh [23, 35] has also been demonstrated. We conclude that in salmon testis tissue, there is also a reduction in Amh activity during recruitment into maturation, reflecting Fsh and/or androgen action.

### Oxidative stress

Up-regulation of the zinc transporter *zip1* (a.k.a. *slc39a1*) transcript in maturing testes is relevant considering that zinc is essential for spermatogenesis in the eel, where it accumulates in the mitochondria of spermatogonia [61]. High levels of copper/zinc superoxide dismutase (Sod1) protein protects eel spermatogonia against reactive oxygen species (ROS)-induced damage [62]. Also in maturing salmon testis, *sod1* transcript levels were up-regulated and *sod1* was retrieved in the functional (KEGG) analysis in the peroxisome pathway (Additional file 5: Table S4). Hence, activating single cell proliferation also activated mechanisms protecting the newly formed cells against ROS-induced damage. This is particularly important in stem cells where ROS-induced DNA damage reduces self-renewal capacity [63].

Several metabolic pathways were functionally enriched in our data set (Fig. 3b), with the large majority of genes being up-regulated (Additional file 5: Table S4). It is reasonable to assume that expansion of the spermatogonial and Sertoli cell populations required additional metabolic efforts. A closer look into these genes touches interesting aspects of stem cell biology. Tissue-specific stem cells in various tissues reside in a hypoxic niche, i.e. the stem cells are exposed to a comparatively low oxygen concentration, and the stem cells moreover are characterized metabolically by elevated gluconeogenesis in combination with active glycolytic and pentose phosphate pathways [64], allowing them to cope with the relative hypoxia. Several genes of all three pathways were up-regulated (Additional file 5: Table S4) in the early maturing salmon testis. In addition, 18 genes of the oxidative phosphorylation pathway were up-regulated, which is not typical for resting or quiescent stem cells. It appears reasonable to assume that a shift from quiescence to increased single cell proliferation activity needs additional metabolic energy. This higher demand for metabolic energy may require a more efficient ATP synthesis, for example by channeling pyruvate into the mitochondrial Krebs cycle for mitochondrial ATP production. This adjustment not only requires up-regulation of oxidative phosphorylation pathway genes, but also increases ROS production [63]. Therefore, one way of understanding the transcriptomic changes regarding metabolic pathways in our data set is to assume that the activation of single cell proliferation generated a population of self-renewing spermatogonia and Sertoli cells. The increased number of these cells may have increased the representation of metabolic pathways typically encountered in stem cells. Moreover, potentially restricted to the fraction of cells actually proliferating at the time of sampling, the newly formed cells may be responsible for the up-regulation of oxidative phosphorylation and ROS production to cope with the elevated metabolic requirements, while reducing the risk of ROS-related DNA damage by increased zinc import and Sod1 levels.

### Wnt signaling

Several members of the Wnt signaling pathway have been retrieved by functional enrichment studies. Many of these genes were related to proteasome activity (Fig. 3a): among the 37 unique genes, 22 genes encoded proteasome building blocks. However, next to breaking down beta-catenin, a critical activator of the canonical Wnt pathway, up-regulation of proteasome activity is associated with proliferation/growth in different tissues, such as skeletal [65] and heart muscle [66] or brain [67], but is also associated with growth in cancer [68]. Therefore, we consider up-regulation of proteasome-associated gene expression as a reflection of the beginning of super-allometric growth of testis tissue in males recruited into maturation. While Wnt ligands or receptors were not retrieved in a prominent fashion, it is interesting to note that the non-canonical ligand *wnt11* and the receptor *fzd8b* (Fig. 3a and Additional file 5: Table S4) were among the DEGs. In mammals, FZD8 is considered to be the receptor binding the non-canonical ligand WNT11 [69], and non-canonical signaling –although involving another non-canonical ligand (Wnt5a)– stimulated self-renewal proliferation of type A_und_ spermatogonia in the adult zebrafish testis [18].

### Caloric restriction and testicular gene expression

To our knowledge, this is the first study investigating the influence of the nutritional status on transcriptomic changes associated with the entry into pubertal testis maturation in fish. As mentioned earlier, RR males showed a lower maturation rate than NR males in the three samplings in December and January (29% vs. 50%; data not shown). Still, maturation occurred frequently also in RR males and, once initiated, proceeded in an apparently similar manner. So we were surprised to see that very many DEGs (887) were retrieved when comparing RR_mat_ with NR_mat_ males (Fig. 2a), also because in adult mice, caloric restriction resulted in very limited changes in testicular gene expression ([70]; less than 20 DEGs). Closer analysis of the salmon data showed that a large fraction (449) of these DEGs displayed low levels of expression in immature testes, significantly elevated expression in NR_mat_ testes, and further up-regulated expression in RR_mat_ testes (Fig. 2d). Importantly, quite similar results were obtained from KEGG pathway and functional enrichment analyses using these two (887 and 449) DEG sets. In other words, initiating testis maturation under caloric restriction required a stronger up-regulation of the same set of 449 DEGs than was necessary in testis tissue from maturing fish receiving ample food.

A more prominent difference between immature and maturing fish in RR than NR males was also observed for other measured reproductive parameters such as 11KT plasma levels and testicular growth factor (*insl3*, *amh, igf3*) transcript levels (Fig. 1). As mentioned earlier, all of these respond to Fsh in fish. We have not generated mechanistic data in the present study. However, considering that also the pituitary transcript levels of *gnrhr4* (1.9-fold vs. 1.4-fold) and *fshb* (7.5-fold vs. 1.9-fold) were up-regulated more clearly in RR_mat_ than in NR_mat_ males (Table 1), we assume that a stronger Fsh stimulation may have contributed to triggering the responses specific to RR_mat_ males. One possible biological explanation for this observation is that with a high energy input, males can support testis maturation with a weaker stimulation from pituitary and testicular hormones, a feature that may serve to avoid negative aspects of high 11KT plasma levels, such as aggressiveness and the resulting danger of injuries, impaired immune system, increased exposure to predators, etc. [71, 72].

Testis tissue of RR_mat_ fish were characterized by a specific set of 421 DEGs (Fig. 2c). Despite this quite high number of DEGs, only three KEGG pathways were retrieved as significantly enriched, the *Metabolic pathways* with 27 DEGs being the largest one (Additional file 6: Figure S2C). For the majority (23) of these genes, information in zebrafish was available on the interaction of this gene set (Fig. 4), and *Carbon* but also *Purine*/*Pyrimidine* metabolism were among the significantly enriched pathways when analyzing gene interaction (Additional file 6: Figure S2D). The relevance of this group of genes involved in carbon metabolism, and therefore their potential role in regulating basic biological processes may explain that many of these 27 genes have been identified previously in zebrafish as modulated in a variety of developmental processes: gastrointestinal tract development (20 out 27; [73]), myogenesis (20 out of 27; [74]) and neural crest/melanoma formation (17 out 27; [75]). In fact, carbon and purine/pyrimidine metabolism are also metabolically intertwined, as carbon metabolism supports nucleotide biosynthesis, but also amino acid homeostasis and epigenetic dynamics [76]. Because of the feeding restrictions, RR_mat_ males are likely to require an elevated testicular production of nucleic acid building blocks when activating testicular cell proliferation. In NR_mat_ males, on the other hand, the building blocks (e.g. purines and pyrimidines) would become available via the diet, limiting additional metabolic efforts of testis tissue. Next to the aspect of providing building blocks for nucleic acid synthesis, two typical intermediates in carbon metabolism are acetyl-CoA, required for histone acetylation, and S-adenosylmethionine (SAM), required as methyl donor for both DNA and histone methyltransferase enzymes [77]. Since changes in the cellular concentrations of acetyl-CoA or SAM are reflected in changes of the epigenetic marking, it is also possible that the observed differences in the magnitude of gene expression can be understood as reflecting an increased activity in writing (mainly activating) epigenetic marks, an interesting hypothesis to be tested in future work.

## Conclusions

We studied the transition from an immature, prepubertal stage to the mitotic expansion of the Sertoli cell and spermatogonial populations in the salmon testis, while providing two different feeding regimes. This allowed retrieving transcriptional networks activated during this initial step of puberty and revealed how their activity was modulated by the energy status. These networks showed many similarities to those identified earlier in studies on pubertal testis development in other vertebrates that, however, covered a much broader range of developmental stages. Besides several signaling systems one would expect to find modulated in pubertal testis tissue (e.g. Tgfβ, Wnt, insulin/Igf, nuclear receptor mediated signaling), we also retrieved metabolic pathways that may be critical for the developmental stage we focused on (i.e. expansion of the spermatogonial stem cell niche), such as the anaerobic metabolism and protection against ROS found in other stem cell systems. Offering two feeding regimes revealed that the transcriptional changes associated with maturation is achieved at lower levels of reproductive hormones and lower transcript levels for a large set of genes when ample food is available. Remarkably, carbon metabolism (involved in nucleotide biosynthesis but also in producing metabolic intermediates required for placing epigenetic marks) was specifically up-regulated in maturing testis tissue from fish reared under caloric restriction.

## Methods

### Animals and experimental design

During September 1–4, 21-month old Atlantic salmon (*N* = 841; body weight 1750 ± 42.4 g, length 51.6 ± 4.0 cm, mean ± SD) were dip-netted from a sea cage (L × W × D, 12 × 12 × 12 m), in which they had been kept since their transfer to seawater at the beginning of May, following smoltification. The fish were randomly distributed between two experimental sea cages (5 × 5 × 8 m) at the Matre Aquaculture Research Station, located in the Matre fjord in Western Norway (61°N). At transfer, the fish were anesthetized (6 ppt metomidate; Syndel, Victoria, B.C.) and individually tagged intraperitoneally with PIT tags, before being measured for body length in cm (BL) and body weight in g (BW).

The fish were maintained under ambient light and were fed with formulated dry feed (Bio optimal, 500, 1000 and 2000 according to fish size, BIOMAR ASA, Norway). One group of fish was fed ad libitum every day (normal ration, NR) during daylight hours using a computer-controlled feeding system, whereas the other group of fish were subjected to a restricted ration (RR; i.e. 43% of the NR ration). This was accomplished by feeding the RR group the same ration as the NR group, but only on Mondays, Wednesdays and Fridays. The approach was based on previous studies in chinook salmon [27], where reduced rations and the resulting ~ 50% lower body weight also reduced the percentage of males entering puberty without affecting survival. Moreover, analyzing the relation between body weight and male maturation in Atlantic salmon smolts showed that body weight is an important factor for increasing the chance to enter puberty [28, 29].

### Sampling

Samples for microarray analysis were collected on December 1, January 5 and 26. At each sampling, 20 fish per group were netted and immediately anaesthetized with 6 ppt metomidate and BL and BW were measured. Blood (5 mL) was collected in heparinized syringes from the caudal vein, and placed on ice. Within 15 min, the blood was centrifuged and the plasma obtained and stored at − 80 °C until analyses. After the blood sampling, the fish were killed by cutting the medulla oblongata, sexed and the pituitary and gonad tissues dissected out. The pituitaries were immediately snap-frozen in aluminum wraps in liquid nitrogen. Gonads were weighed (GW, g) for gonadosomatic index (GSI) determination (GSI = GW × 100 × BW^− 1^). The condition factor (K) was calculated as K = BW × BL^− 3^ × 100. Tissue pieces were obtained from the cranial third of the testis, by transversal cuts with a scalpel. One piece was collected for histological analysis as described below, while another piece was snap-frozen in aluminum wraps in liquid nitrogen. Viscera (including associated adipose tissue but excluding liver/gall bladder) and a sample from the dorsal skeletal muscle tissue were dissected out, weighed and stored at − 20 °C until dry matter and lipid analysis, as described previously [78]. In brief, the procedure involved homogenization, determination of dry matter, followed by the extraction and weighing of the total lipids. Results are expressed as % total lipid content of the dry matter.

### Selection of males for microarray analysis

To select individuals for microarray analysis, testis tissue samples were analyzed histologically for the cellular composition and the proliferation activity of Sertoli and germ cells. To this end, testes were fixed in Bouin’s fluid, embedded in paraffin and sectioned at 5 μm thickness, according to conventional techniques. Sections were stained with hematoxylin/eosin combined with periodic acid according to Schiff (H/E-PAS) and scored for the most advanced stage of spermatogenic development, differentiating between spermatogonia type A and type B, as described previously [26]. Germ cells developed beyond the stage of type B spermatogonia were not recorded in the testis sampled collected until January 26 (Additional file 1: Figure S1).

Other sections were used for the immunocytochemical detection of phosphorylated histone H3 (pH3), a marker for proliferating cells, as described previously [79], except that the primary antibody was detected by undiluted HRP-conjugated goat anti-rabbit IgG (Brightvision Immunologic, Duiven, The Netherlands). Previous studies on the analysis of testicular proliferation in Atlantic salmon post-smolts showed that males before and just after the start of testis development can be clearly separated based on the proliferation activity [26]. The sections were scored semi-quantitatively for the incidence of proliferating cells (Additional file 1: Figure S1). With type A spermatogonia as the most advanced germ cell type and up to 3 single, pH3-positive (germ or Sertoli) cells found per view field on 5 view fields at 400-fold magnification, the individual was scored pre-pubertal. When type A spermatogonia were the most advanced germ cell type but 6 or more single (germ or Sertoli) cells were found pH3-positive, the individual was scored as pubertal.

Morphological parameters formed the primary basis for differentiating between immature and maturing males, but we also analyzed a selection of other recognized reproductive parameters: plasma 11-ketotestosterone (11KT) levels were quantified by radioimmunoassay as described previously [80]; qPCR (real-time, quantitative polymerase chain reaction) was used to quantify the expression of selected genes in the pituitary (*fshb*, *lhb*, and *gnrhr4*; as described previously by Melo and co-authors [26]), or in the testis (*igf3*, *insl3* and *amh*), for which specific qPCRs were developed (Table 2). These growth factor genes are known to show significant changes in testicular gene expression during spermatogenesis in different fish species (Igf3, [20]; Insl3, [17]; Amh, [19, 44, 81]).

**Table 2:** Primers used for gene expression studies by qPCR analysis

Gene description	Target gene	Primers	Primer sequence (5′ → 3′)
Anti-Müllerian hormone	*amh*	Fw	CAGTCACTCTCTGCAGCCTTACAA
		Rv	CAACATTGAATCTCCATTTCAGTTTAC
		Probe	TTTGCCCTCGGGTTGCTTTCCTGTT
Calreticulin precursor	*calr*	Fw	ACGCCGCTGACTCTACCATCT
		Rv	TCACATCATCTGAGACCAGGAAGTT
Catenin beta-1	*ctnnb1*	Fw	CCCCAGGCGACAGCAAT
		Rv	CCAAATCACAATGCAGGTTGA
Cryptochrome-1	*cry1*	Fw	AGGTCAGCCGGCCAACA
		Rv	AACGGCTCAGAGTCACACTCAAA
Endoplasmin precursor	*hsp90b1*	Fw	TGAAGCGGATTTGGTTTATTGG
		Rv	CGCCGTCAATGTCAAGTTCAT
Eukaryotic 18S ribosomal RNA	*18S*	TaqMan^a^	proprietary information
Follicle stimulating hormone subunit beta	*fshb*	Fw	TCACGGAGGCATCACCATCA
		Rv	GCTCTTGGCAACGGGTATGA
		Probe	ACCTGCGCCGGCCTGTGC
Frizzled-8 precursor	*fzd8l*	Fw	GGAGCGACCCATTATTTTCCT
		Rv	CCGGCAATTAGTCTGACAATGTAA
G1/S-specific cyclin-D1	*ccnd1*	Fw	AAGCAAATCCTGTGCAAGCA
		Rv	CGGAGGGTTGGCAATGAA
Gamma-crystallin M2	*crygm2*	Fw	CCACCTTCTACGAGGACAGGAA
		Rv	TGGCACCTGCTCATGTAGGA
Gap junction alpha-1 protein	*gja1*	Fw	CCTAAGGGCTCTCTTCTTACTTCTGA
		Rv	CACTCCAGTCACCCATGATGAA
Geminin	*gmnn*	Fw	TGCAAGTCCTCCAGCAAGCT
		Rv	CCCTTGTTCTGCATTCCACTGT
Gonadotropin releasing hormone receptor 4	*gnrhr4*	Fw	TCAACCCACTGGCGATCAAT
		Rv	CGTGATGGTCACACTGTGGAATA
		Probe	AGTGTGATTCTGTCTGTTCCCCAGATGCTG
Gonadotropin subunit beta-2	*lhb*	Fw	ACACTGCCCATCAGGACACAA
		Rv	GGGAGATCAAGGTGCCTACATG
Growth-regulated alpha protein-like	LOC106577834	Fw	TCATCATGAATACTGCAATGACTGTT
		Rv	TATCCTCTCCCTTGTATCATGCAGAT
Insulin-like 3 (Leydig cell)	*insl3*	Fw	CTCCGGAGCTTGGACAACAC
		Rv	AGTCCTCAGGTTGGCAAATTGAT
Insulin-like growth factor 3	*igf3*	Fw	GACCGACCGACAAGATGCA
		Rv	TGCAAGGCACAATATGGAGTACA
Noggin	*nog*	Fw	GATGGAGGTGCCTGCAGAGA
		Rv	TTCAGTTCGAGCAGGAGCATTT
Olfactomedin-like protein 2B precursor	*olfml2b*	Fw	CGAAGGGCAACGGAAATGTA
		Rv	ATCCAGTTGTACGGCAGCTTGT
Prostaglandin E synthase 3	*ptges3*	Fw	CCGTGGCTGAGGCTAACAAA
		Rv	CCTCCCAGTCTTTCCAGTTGTTAA
Testin	*tes*	Fw	CGTGTCTGAAATGCAAGGACAA
		Rv	GTGGTCCGTAAGCCCACACTT
Vasotocin-neurophysin VT 2 precursor	*neu4*	Fw	GGACACTGCGCTGCAACA
		Rv	GTACCGACTCTCCCCCAAACA
Wnt-11 precursor	*wnt11l*	Fw	CCCCAGGCCAGCACACTA
		Rv	ACCACCTGCAGAGCCTCACT

^a^20X eukaryotic *18S* rRNA pre-developed TaqMan assay (4352930E; Applied Biosystems). *Fw* Forward, *Rv* Reverse

Hence, males were classified into four groups for the transcriptomic analyses (see below), reflecting the feeding regime (NR or RR) and immature (≤ 3 proliferating single cells) or maturing males (≥ 6 proliferating single cells), respectively. The groups are referred to as NR_imm_, NR_mat_, RR_imm_ and RR_mat_.

### Transcriptomic analyses

Testicular tissue was homogenized in 2 mL tubes containing Trizol reagent from the iPrep Trizol Plus RNA Kit (Invitrogen, Carlsbad, California, USA) and zirconium oxide beads in a Precellys 24 Homogenizer (Bertin, Villeurbanne, France). Subsequently, total RNA was extracted in an iPrep Purification Instrument (Invitrogen), according to the manufacturer’s instructions. Total RNA yield was determined using a NanoDrop ND-1000 spectrophotometer (Labtech, Palaiseau, France) and RNA integrity was checked with an Agilent Bioanalyzer (Agilent Technologies, Massy, France). Gene expression profiling was conducted using an Agilent 4x44K cGRASP high-density oligonucleotide microarray (GPL11299; AMADID 025055; http://web.uvic.ca/grasp/microarray/array.html; [82]). The array contained 16 K unique genes, representing ~ 45% of the Atlantic salmon genome. Labeling and hybridization steps were performed according to the Low Input Quick Amp Labeling Kit protocol (One-Color; Agilent Technologies). For each sample, 150 ng of total RNA was amplified and labeled using Cy3-CTP. Yield (> 0.825 μg cRNA) and specific activity (> 6 pmol of Cy3 per μg of cRNA) of Cy3-cRNA produced were checked with NanoDrop, and 600 ng of each Cy3-cRNA was fragmented and hybridized on a different subarray. Hybridization was carried out for 17 h at 65 °C in a rotating hybridization oven prior to washing and scanning (DNA Microarray Scanner; Agilent Technologies) using standard parameters (5 μm and 20 bits). Data were then collected with Feature Extraction software (version 10.7.1.1; Agilent Technologies), log2-transformed and submitted to a quantile normalization. The complete raw data have been deposited in the NCBI GEO database under accession number GSE126582. Focusing on maturation effects we compared all immature vs. all maturing. Focusing on feed ration effects we first compared all NR vs. all RR, and due to the low number of differentially expressed genes (DEGs) (38) we continued by comparing NR_imm_, with RR_imm_ and NR_mat_ with RR_mat_. Normalized and filtered data were imported into GeneSpring GX version 11.0.2 (Agilent Technologies). To assess genes for differential expression, log2-transformed intensity ratios were analyzed using Student’s t-test (fold change > │1.5│; *P* < 0.01 at the α level of 0.05, adjusted by Benjamini-Hochberg correction).

Sets of modulated genes were compared using Venn diagrams (Venny 2.0 software; http://bioinfogp.cnb.csic.es/tools/venny/index.html) to identify common and specific DEGs between comparisons. Regulated KEGG pathways were determined using the KEGG Mapper tool [83]. KEGG pathways represented by at least 5 DEGs and by the ratios of regulated genes (up−/down-, and vice versa) higher than 5 were considered for the analysis. Functional enrichment analyses were performed using a freely available plugin (http://www.baderlab.org/Software/EnrichmentMap) [84] for the Cytoscape network environment [85]. Enrichment Map plugin calculates over-representation of genes involved in closely related Gene Ontology (GO) categories [86], resulting in a network composed by gene sets associated and grouped according to their function. DAVID Bioinformatics Resources 6.7 [87] was used to retrieve GO terms (biological process level) from the list of DEGs and exported as the input. For both (i.e. KEGG and enrichment map) analyses, human orthologues of the DEGs were used as input.

### Protein-protein interaction analysis

STRING database v10.5 (http://string-db.org/; [88]) was used for protein-protein interaction analysis among identified genes (see Fig. 4), which groups proteins based on pairwise similarities in relevant descriptor variables, resulting in functionally connected network sets. A FDR test was performed to determine if the protein list was enriched in interactions.

### Studies on candidate gene expression and validation

The relative transcript levels of selected genes of interest (Figs. 1 and 4) and genes for microarray validation (Fig. 5) were studied by qPCR using specific primer sets (see Table 2 for detailed primer information). For this purpose, aliquots of the sample set used for microarray hybridization were also analyzed as previously described [34, 89]. 18S rRNA (commercially available TaqMan gene expression assay; Applied Biosystems) was used as the housekeeping control gene due to its stable expression in all sample groups analyzed.

### Statistical analysis

GraphPad Prism 5.0 package (GraphPad Software, Inc.) was used for statistical analysis. Significant differences between groups were identified using Student’s t test, one-way ANOVA followed by Tukey’s test, or two-way ANOVA followed by Bonferroni’s test for multiple group comparisons, respectively. Data are presented as means ± SEM. Correlation of qPCR data with microarray-derived data was tested using Spearman’s rank test. *P* values were tested against the critical value of α < 0.05 and when *P* values were smaller than that (*P* < 0.05), they were considered significant.

## Additional files

Additional file 1: **Figure S1.** Morphological evaluation of the maturational status in male salmon. Representative histology (hematoxylin/eosin combined with periodic acid according to Schiff, H/E-PAS; **(A-C)**) and proliferation activity (immune-detection of phosphorylated histone H3, pH 3; **(D-F)**) in testes tissue obtained from Atlantic salmon in different stages of maturation. Representative type A undifferentiated (black arrowheads), type A differentiating (black dashed lines), type B spermatogonia (white dashed lines), and “free” Sertoli cells (black arrows) are shown in B-C. In E-F, representative pH 3-positive cells are indicated by red arrowheads (type A undifferentiated spermatogonia), red dashed lines (type B spermatogonia) and red arrows (“free” Sertoli cells), respectively. Scale bar represents 25 (A) or 30 (B-F) μm. (TIF 6792 kb)

Additional file 2: **Table S1.** Influence of the feed ration, or the maturational status, on selected morphometric parameters, pituitary gene expression, and plasma hormone levels. RF, restricted ration; NF, normal ration; GSI, gonadosomatic index; K, condition factor; RU, relative units; nd, not determined. In the two different comparisons (ration effect and maturation effect; highlighted by different background color), asterisks indicate significant differences (*N* = 5–16; Student’s t-test, * *P* < 0.05 at the α level of 0.05) between groups. (DOCX 20 kb)

Additional file 3: **Table S2.** Complete microarray results showing modulated testicular gene expression of immature and maturing Atlantic salmon fed a normal (NR) or restricted ration (RR; 43% of NR). Excel file (.xlsx) containing normalized expression data, statistics, and gene description and regulation, for all differentially expressed genes (*N* = 5–16; Student’s t-test, *P* < 0.01 at the α level of 0.05, FDR < 0.05; fold change > │1.5│) identified in all 4 comparisons described in this study. (XLS 483 kb)

Additional file 4: **Table S3.** Common differentially expressed genes identified in both all maturing salmon and maturing salmon exposed to restricted feeding conditions. Excel file (.xlsx) containing in the first sheet normalized expression data, and gene description and regulation, for all shared differentially expressed genes (449; Student’s t-test, *P* < 0.01 at the α level of 0.05, FDR < 0.05; fold change > │1.5│) identified in both pairwise comparisons. The second data sheet shows, for all shared genes, the magnitude of changes in expression in different experimental conditions. (XLS 163 kb)

Additional file 5: **Table S4.** Detailed KEGG pathway information. Each pathway shown is represented by at least 5 DEGs (*P* < 0.01 at the α level of 0.05, FDR < 0.05) and has a ratio of regulated genes (up−/down-, or vice versa) higher than 5. DEGs are highlighted with red (up-) or green (down-regulated) background. (XLSX 23 kb)

Additional file 6: **Figure S2.** KEGG pathways identified in the maturing salmon testis in response to the food ration. **(A)** Specific KEGG pathways found exclusively in maturing males exposed to the normal ration. **(B)** Common KEGG pathways observed in all maturing salmon independently of the feed ration. **(C)** Specific KEGG pathways identified exclusively in maturing males exposed to the restricted ration. Each pathway shown is represented by at least 5 DEGs (*P* < 0.01 at the α level of 0.05, FDR < 0.05) and has a ratio of regulated genes (up−/down-, or vice versa) higher than 5. DEGs are highlighted with red (up-) or green (down-regulated) background. **(D)** Complete list of pathway IDs retrieved from the 23 interacting proteins identified in Fig. 4 (*P* < 1.0E-16 at the α level of 0.05, FDR < 0.05). (TIF 17596 kb)

Additional file 7: **Table S5.** Androgen-induced changes in salmonid testicular gene expression. List of potentially modulated genes identified in both salmon (this study) and rainbow trout testes [48]. (XLSX 9 kb)

## Data Availability

The complete raw RNAseq data have been deposited in the NCBI GEO database under accession number GSE126582. Analyzed and filtered data are available in the article and its supplementary information files.

## Abbreviations

11KT: 11-ketotestosterone
A_diff_: Type A differentiating spermatogonia
Amh: Anti-Müllerian hormone
A_und_: Type A undifferentiated spermatogonia
BL: Body length
Bmp: Bone morphogenetic protein
BW: Body weight
DEG: Differentially expressed gene
FDR: False discovery rate
Fsh: Follicle-stimulating hormone
Gnrh: Gonadotropin-releasing hormone
GO: Gene ontology
GSI: Gonadosomatic index
hCG: Human chorionic gonadotropin
HRP: Horseradish peroxidase
Igf: Insulin-like growth factor
Insl3: Insulin-like 3
K: Condition factor
KEGG: Kyoto encyclopedia of genes and genomes
Kiss: Kisspeptin
Lh: Luteinizing hormone
NR: Normal ration
NR_imm_: Normal ration immature
NR_mat_: Normal ration maturing
pH 3: Phosphorylated histone H3
qPCR: Quantitative polymerase chain reaction
ROS: Reactive oxygen species
RR: Restricted ration
RR_imm_: Restricted ration immature
RR_mat_: Restricted ration maturing
SSC: Spermatogonial stem cell
Tgfβ: Transforming growth factor beta
Wnt: Wingless-type MMTV integration site
