# Supplementary figures and images for: The initiation of puberty in Atlantic salmon brings about large changes in testicular gene expression that are modulated by the energy status
Source: BMC Genomics. 2019 Jun 11;20:475. doi: 10.1186/s12864-019-5869-9 (PMC6558769; doi:10.1186/s12864-019-5869-9)

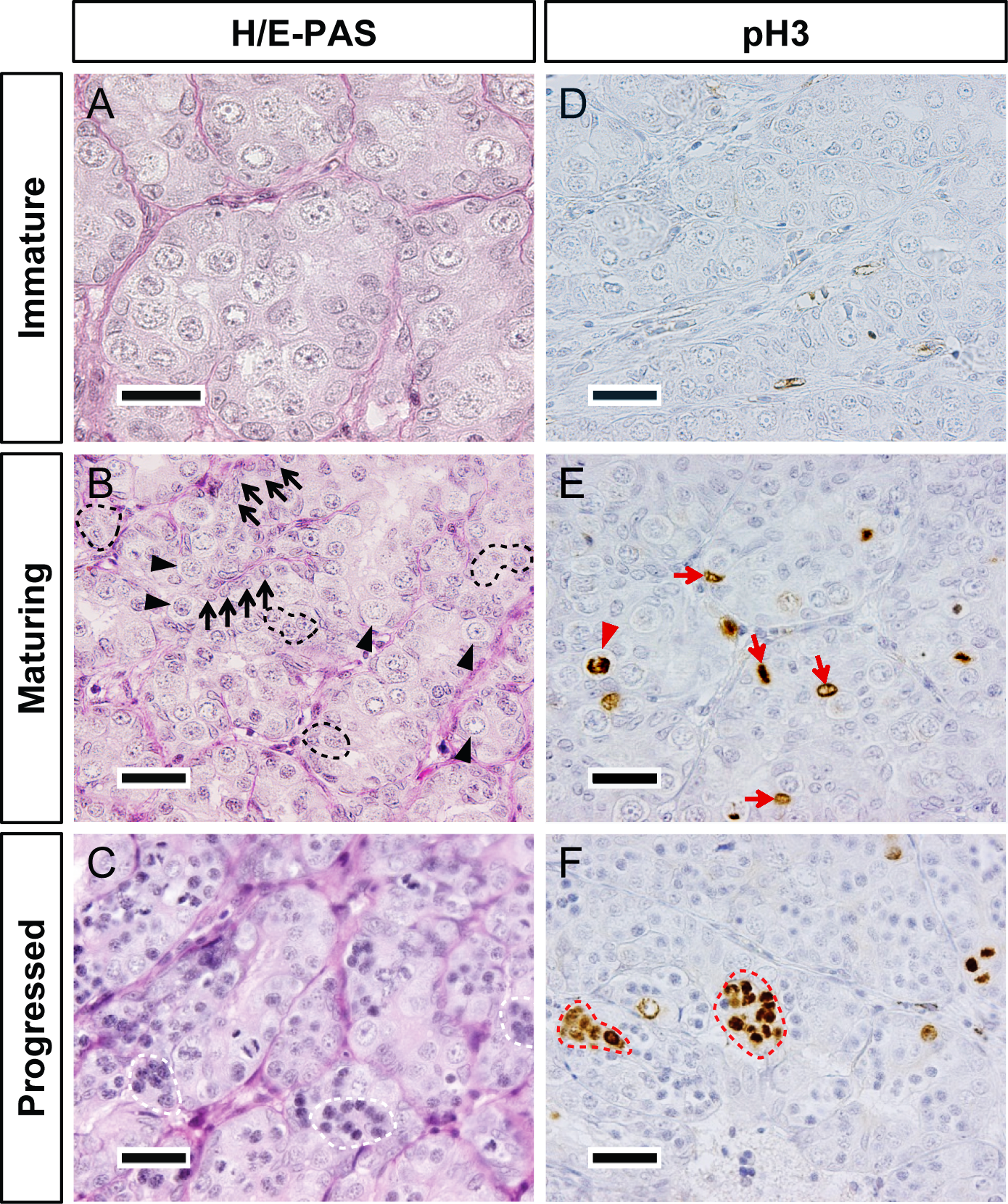

Supplement: Supplementary file 1 — Figure S1. Morphological evaluation of the maturational status in male salmon. Representative histology (hematoxylin/eosin combined with periodic acid according to Schiff, H/E-PAS; (A-C)) and proliferation activity (immune-detection of phosphorylated histone H3, pH 3; (D-F)) in testes tissue obtained from Atlantic salmon in different stages of maturation. Representative type A undifferentiated (black arrowheads), type A differentiating (black dashed lines), type B spermatogonia (white dashed lines), and “free” Sertoli cells (black arrows) are shown in B-C. In E-F, representative pH 3-positive cells are indicated by red arrowheads (type A undifferentiated spermatogonia), red dashed lines (type B spermatogonia) and red arrows (“free” Sertoli cells), respectively. Scale bar represents 25 (A) or 30 (B-F) μm. (TIF 6792 kb) [file 12864_2019_5869_MOESM1_ESM.tif]

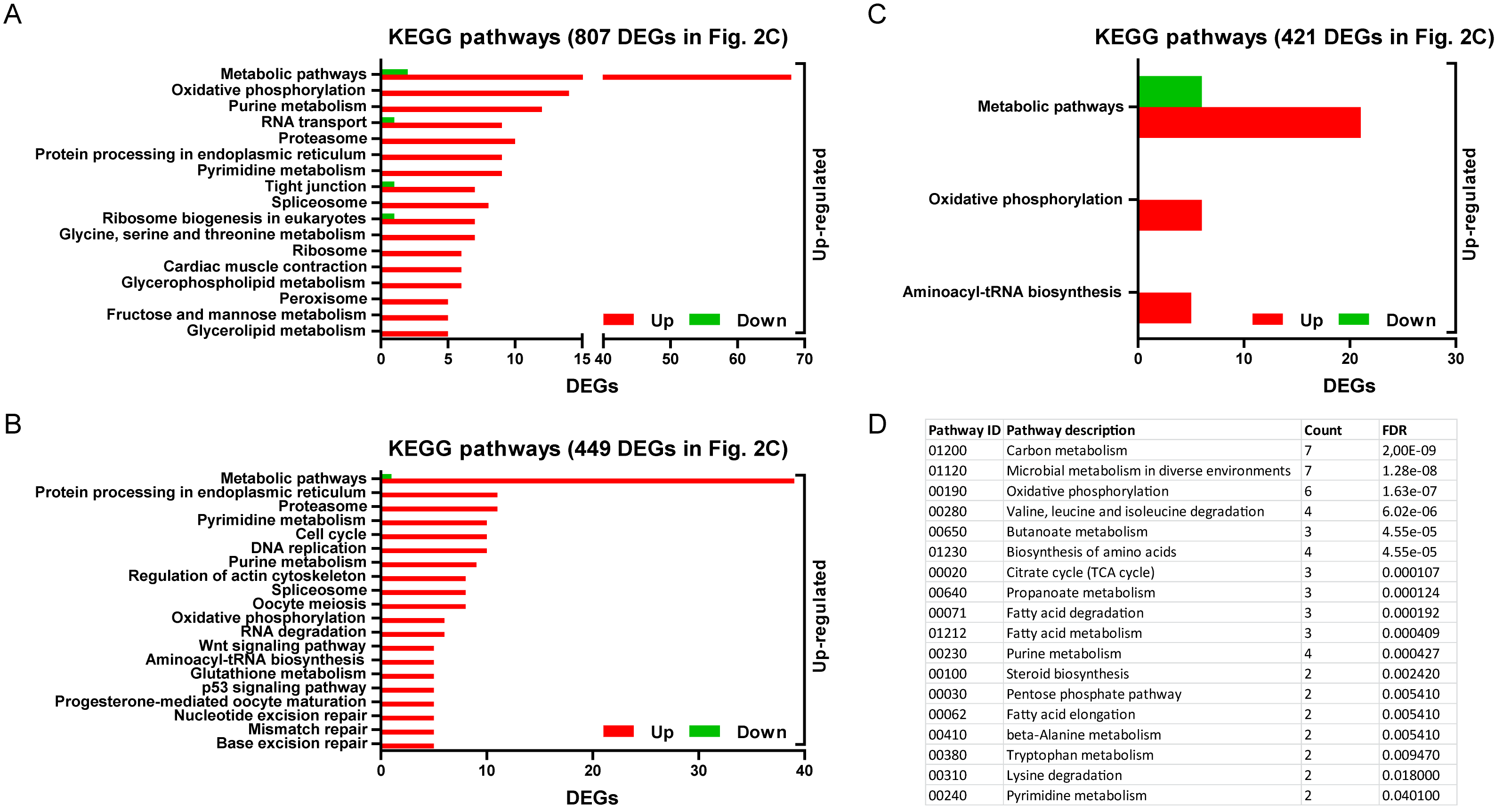

Supplement: Supplementary file 6 — Figure S2. KEGG pathways identified in the maturing salmon testis in response to the food ration. (A) Specific KEGG pathways found exclusively in maturing males exposed to the normal ration. (B) Common KEGG pathways observed in all maturing salmon independently of the feed ration. (C) Specific KEGG pathways identified exclusively in maturing males exposed to the restricted ration. Each pathway shown is represented by at least 5 DEGs (P < 0.01 at the α level of 0.05, FDR < 0.05) and has a ratio of regulated genes (up−/down-, or vice versa) higher than 5. DEGs are highlighted with red (up-) or green (down-regulated) background. (D) Complete list of pathway IDs retrieved from the 23 interacting proteins identified in Fig. 4 (P < 1.0E-16 at the α level of 0.05, FDR < 0.05). (TIF 17596 kb) [file 12864_2019_5869_MOESM6_ESM.tif]
