# Supplementary material for: The initiation of puberty in Atlantic salmon brings about large changes in testicular gene expression that are modulated by the energy status
Source: BMC Genomics. 2019 Jun 11;20:475. doi: 10.1186/s12864-019-5869-9 (PMC6558769; doi:10.1186/s12864-019-5869-9)
Supplement: Supplementary file 2 — Table S1. Influence of the feed ration, or the maturational status, on selected morphometric parameters, pituitary gene expression, and plasma hormone levels. RF, restricted ration; NF, normal ration; GSI, gonadosomatic index; K, condition factor; RU, relative units; nd, not determined. In the two different comparisons (ration effect and maturation effect; highlighted by different background color), asterisks indicate significant differences (N = 5–16; Student’s t-test, * P < 0.05 at the α level of 0.05) between groups. (DOCX 20 kb) [file 12864_2019_5869_MOESM2_ESM.docx]

**Additional file 2: Table S1. Influence of the ration regime, or the maturational status, on selected morphometric parameters, pituitary gene expression, and plasma hormone levels.**

|  | RR | NR | Immature | Maturing |
| --- | --- | --- | --- | --- |
| 11KT (ng/mL) | 1.74 ± 0.48 | 1.68 ± 0.30 | 0.79 ± 0.08 | 4.20 ± 0.58* |
| Weight (Kg) | 2.45 ± 0.13 | 4.33 ± 0.32* | 2.91 ± 0.20 | 4.21 ± 0.50* |
| Length (cm) | 59.57 ± 1.13 | 66.75 ± 1.30* | 61.07 ± 1.08 | 67.00 ± 1.91* |
| Visceral fat (%) | 24.74 ± 1.54 | 36.84 ± 0.95* | 31.09 ± 1.60 | 38.10 ± 1.79* |
| Muscle fat (%) | 14.18 ± 0.38 | 17.94 ± 0.57* | 15.44 ± 0.46 | 19.68 ± 0.61* |
| GSI (%) | 0.07 ± 0.01 | 0.08 ± 0.01 | 0.07 ± 0.00 | 0.10 ± 0.01* |
| K | 1.14 ± 0.03 | 1.41 ± 0.04* | 1.24 ± 0.04 | 1.32 ± 0.07 |
| *fshb* (RU) | 10.52 ± 2.97 | 14.33 ± 2.35 | 7.05 ± 1.12 | 25.94 ± 4.30* |
| *lhb* (RU) | 29.82 ±11.10 | 27.88 ± 7.92 | 15.89 ± 4.57 | 64.34 ± 19.72* |
| *gnrhr4* (RU) | 2.09 ± 0.20 | 2.68 ± 0.26 | 1.97 ± 0.12 | 3.34 ± 0.36* |

RF, restricted ration; NF, normal ration; GSI, gonadosomatic index; K, condition factor; RU, relative units; nd, not determined. In the two different comparisons (ration effect and maturation effect; highlighted by different background color), asterisks indicate significant differences (N = 5-16; Student’s t-test, * *P* < 0.05 at α the level of 0.05) between groups.
